# Anti-tumor Study of Chondroitin Sulfate-Methotrexate Nanogels

**DOI:** 10.1186/s11671-017-2324-1

**Published:** 2017-10-24

**Authors:** Jinyu Wang, Weibo Zhao, Haixiao Chen, An Qin, Peizhi Zhu

**Affiliations:** 1 College of Chemistry and Chemical Engineering, Yangzhou University, Yangzhou, 225200 Jiangsu People’s Republic of China; 2 Orthopaedic Department, Taizhou Hospital, Wenzhou Medical University, Taizhou, 318000 Zhejiang, People’s Republic of China; 3 Department of Orthopedic Surgery, Shanghai Key Laboratory of Orthopedic Implants, Shanghai Ninth People’s Hospital, Shanghai Jiao Tong University School of Medicine, Shanghai, China

**Keywords:** Methotrexate, Chondroitin sulfate, Self-assembly, Nanogels, Anti-tumor, Nanomedicine, Nanotechnology

## Abstract

Self-assembly nanogels (NGs) were formed by bioconjugating methotrexate (MTX) with chondroitin sulfate (CS). MTX-CS NGs can greatly enhance the solubility and improve the delivery efficacy of MTX due to the CD44 binding property of CS. Vivo experiments revealed that MTX-CS NGs showed less toxicity than MTX. MTX-CS NGs can improve the anti-tumor effect while reducing the side effects of MTX. Due to their CD44 binding property, chondroitin sulfate-drug conjugates could be a promising and efficient platform for improving the solubility of sparingly soluble drug molecules as well as targeted delivery to cancer cells and tumor tissues.

## Background

Methotrexate (4-amino-10-methylfolic acid, MTX) is a folate analog, belonging to the antifolate antimetabolite family [1]. MTX was the first drug used in tumor therapy from the 1950s [2], which is a mutagen and teratogenic anti-tumor drug, acting by blocking the enzyme activity and interfering with DNA synthesis [3]. Previous studies have shown that the delivery of chemotherapy drugs to the target cell alone is not sufficient to induce cell death, and high-dose MTX can significantly improve the cure rate and the prognosis of patients [4]. Low water solubility, low permeability, and short half-life of MTX limit its clinical application [5, 6]. The effect of chemotherapy of MTX is largely influenced by its low tumor cell uptake, tissue biodistribution, and serious side effects [7]. However, a higher concentration of MTX can increase the risk of adverse effects because of MTX’s poor bioavailability [8]. There is an urgent need to develop a new drug delivery system to improve the bioavailability of MTX and reduce its side effects.

Nanotechnology has advantages in drug delivery systems, including improving the drug stability, extending blood circulation, reducing side effects, and controlling drug release [9–15]. Self-assembly technology has been widely used in the field of drug delivery to enhance the efficacy and lower the adverse effects of drugs [16–20]. Our study aims to design a nanogel drug delivery system for MTX to improve its solubility and biodistribution and reduce its side effect. Chondroitin sulfate (CS) is an acidic glycosaminoglycan (GAG), which constitutes an important component of cartilage, blood vessel walls, skin, tendons, and other connective tissues [21]. Nanogels based on chondroitin sulfate have been studied previously [22, 23]. Studies found that CD44 binds a CS proteoglycan [24–26]. CD44 is a transmembrane glycoprotein with extracellular domains and has been implicated in mediating cell-cell and cell-ECM interactions and plays a role in cell migration [27]. CD44 is highly expressed in metastatic cancer in contrast to its low expression levels in normal tissues [28]. Nanoparticles based on CS have been reported for tumor targeting and anti-tumor drug delivery [29, 30]. Here, we fabricated a novel type of self-assembly CS-MTX nanogels in an effort to enhance the targeted delivery of MTX drug molecules to cancer cells through CS-CD44 interaction.

## Methods

### Materials and Samples

Chondroitin sulfate was purchased from Dalian Meilun Biotech Co., Ltd. (Dalian, Liaoning, China). 4-Methylmorpholine, tetrahydrofuran, and 2-chloro-4, 6-dimethoxy-1, 3, 5-triazine were purchased from Sun Chemical Technology (Shanghai, China) Co., Ltd., and fetal bovine serum (FBS) was purchased from HyClone (Utah, USA). All the other chemicals were purchased from Sinopharm Chemical Reagent Co., Ltd. (Shanghai, China). The Lewis rats were purchased from Shanghai Sippr-BK Laboratory Animal Co., Ltd. (Shanghai, China).

### Synthesis of DMT-MM

**Scheme 1:**
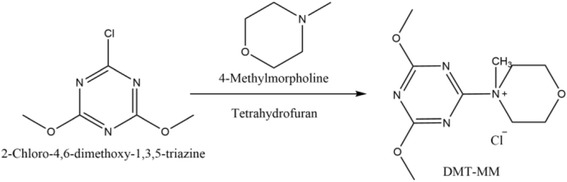
Synthetic pathways in formation of DMT-MM

4-(4,6-Dimethoxy-1,3,5-triazin-2-yl)-4-methylmorpholinium chloride (DMT-MM) is used for dehydration condensation reactions with carboxylic acid activation which can be used in aqueous or proton solvent systems. Twenty-five grams of 2-chloro-4, 6-dimethoxy-1, 3, 5-triazine (CDMT) was dissolved in 200 ml tetrahydrofuran (THF). Then, 18.79 ml of 4-methylmorpholine (NMM) was added dropwise to the CDMT solution under stirring. In order to ensure complete response, stirring should be maintained for 30 min. Then, the filtered product was washed three times by THF and drying under vacuum for 24 h. The DMT-MM was obtained as a white powder (Scheme 1).

### Synthesis of MTX-CS

**Scheme 2:**
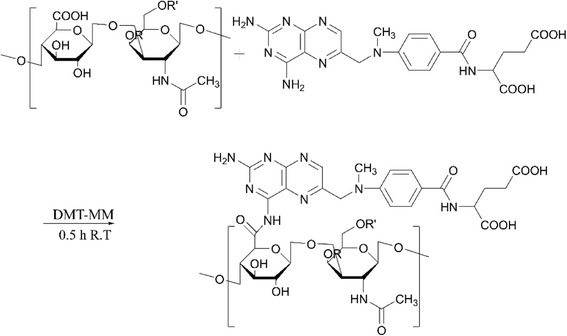
Synthetic pathways in formation of MTX-CS

The MTX-conjugated CS was activated by DMT-MM. For CS activation, CS (1.0 g) was dissolved in 20 ml ultrapure water and activated by adding DMT-MM (0.769 g). The reaction was conducted for 30 min at room temperature. Then, the activated CS was further reacted with MTX for 24 h at room temperature. The solution was dialyzed for 48 h with changing water every 4 h and lyophilized. The MTX-CS was obtained as a yellow powder. The yellow powder was examined by Fourier transform infrared spectrometry (ALPHA, BRUKER, USA). The FTIR spectra were recorded from 400 to 4000 cm^−1^. ^1^H NMR was used to determine whether the MTX was conjugated to CS (Scheme 2).

### Cytotoxicity of MTX-CS Nanogels

The cytotoxicity of the nanogels was analyzed by using A549T and Hela tumor cell and Human Umbilical Vein Endothelial Cell (HUVEC) culture. The A549T and Hela cells were seeded in 96-well plates at a density of 5 × 10^3^ cells per well in 1640, supplemented with 10% FBS and incubated for 24 h under 5% CO_2_ at 37 °C. The A549T was followed by treatment with different concentrations of MTX-CS NGs (0, 5, 10, 20, 30, 40, 50, 100, 200, 400 μM), and the Hela was followed by treatment with different concentrations of MTX-CS NGs (0, 5, 10, 30, 40, 60, 80, 100 μM) for another 48 h. The concentrations of MTX-CS NGs were based on the content of MTX in each sample. The concentrations of CS were based on the content of MTX-CS NGs in each sample. The HUVECs were seeded in 96-well plates at a density of 5 × 10^3^ cells per well in DMEM, supplemented with 10% FBS and incubated for 24 h under 5% CO_2_ at 37 °C. The HUVEC was then added different concentrations of MTX-CS NGs (0, 5, 10, 20, 30, 40, 50, 100, 200, 400 μM). The concentrations of MTX-CS NGs were based on the content of MTX in each sample. The concentrations of CS were based on the content of MTX-CS NGs in each sample. The MTT assay is a measurement of cell activity. Twenty microliters of CCK-8 buffer was added to each well and incubated at 37 °C under 5% CO_2_ for another 4 h. The medium was removed, and 200 μL DMSO was added to each well. The absorbance was measured at a wavelength of 490 nm (570 nm as reference) on a MULTISKAN GO Microplate reader (Thermo Scientific, USA).

### Animal and Experimental Design

In order to analyze the toxicity of MTX-CS NGs in vivo, eighteenth male Sprague-Dawley rats were purchased from the Experimental Animal Center of the Zhejiang Academy of Medical Sciences (Hangzhou, Zhejiang, China). Those rats were housed under a 12-h light, 12-h dark cycle with free access to water and food. The rats, aged 8 weeks (200 ± 10 g), were randomly divided into three groups: control group (injected with the same volume saline), MTX group (injected with 1.25 μmol kg^−1^ day^−1^), and MTX-CS NG group (injected with 25 mg kg^−1^ day^−1^ MTX-CS NGs). The MTX dose of the MTX-CS NG group was equal to a free dose of the MTX group (1.25 μmol kg^−1^ day^−1^). Drugs were given on another day by intraperitoneal injections respectively. After 2 weeks of treatment (total seven injections), all rats were killed by decapitation for further research.

### Histological Study

After decapitation, all rats’ spleens were dissected out rapidly and washed twice with phosphate-buffered saline (PBS) and fixed in 4% (*w*/*v*) paraformaldehyde (pH = 7.4) (Sigma-Aldrich, MO, USA) for 24 h. Then, tissues were prepared for hematoxylin and eosin (H&E) staining using standard procedures and obtained under a high-quality light microscope.

## Results and Discussion

### Synthesis of MTX-CS

**Fig. 1:**
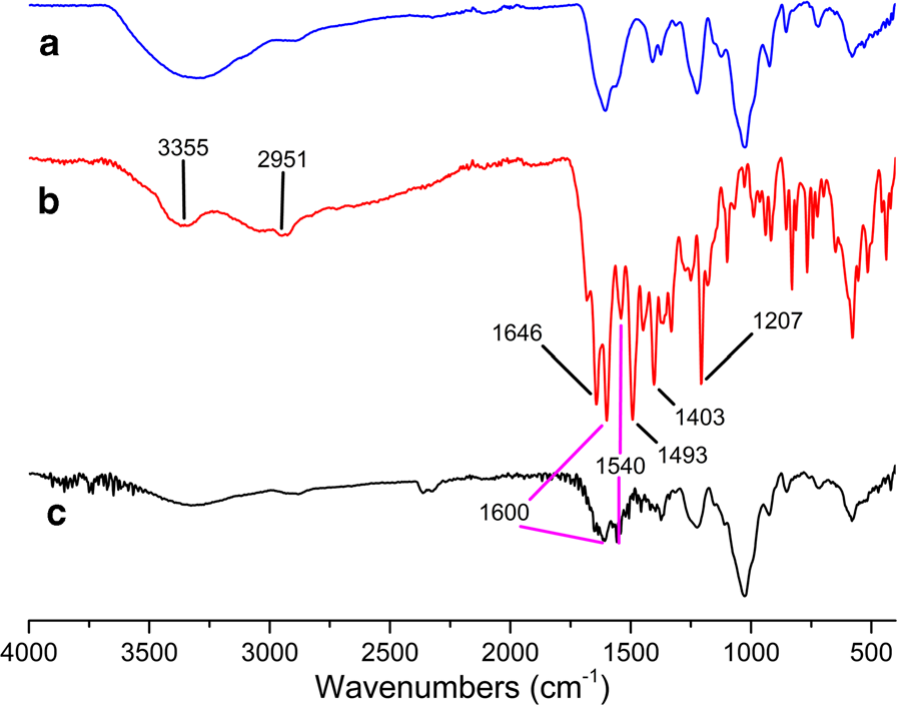
**a** FTIR spectrum of CS. **b** FTIR spectrum of MTX. **c** FTIR spectrum of MTX-CS

To determine whether the MTX was conjugated to CS, we used FTIR and ^1^H NMR to analyze the MTX, CS, and MTX-CS bioconjugate samples. Figure 1 shows the FTIR spectra of CS (Fig. 1a), MTX (Fig. 1b), and MTX-CS bioconjugates (Fig. 1c). As shown by Fig. 1b, MTX had characteristic transmittance at 3355, 2951, 1646, 1600, 1540, 1493, 1403, and 1207 cm^−1^. The FTIR peaks at 1600 and 1540 cm^−1^ can be assigned to stretching of para-benzene, which could be found in the FTIR spectrum of MTX (Fig. 1b) and MTX-CS bioconjugates (Fig. 1c). The FTIR results indicated that MTX was successfully conjugated to CS.

**Fig. 2:**
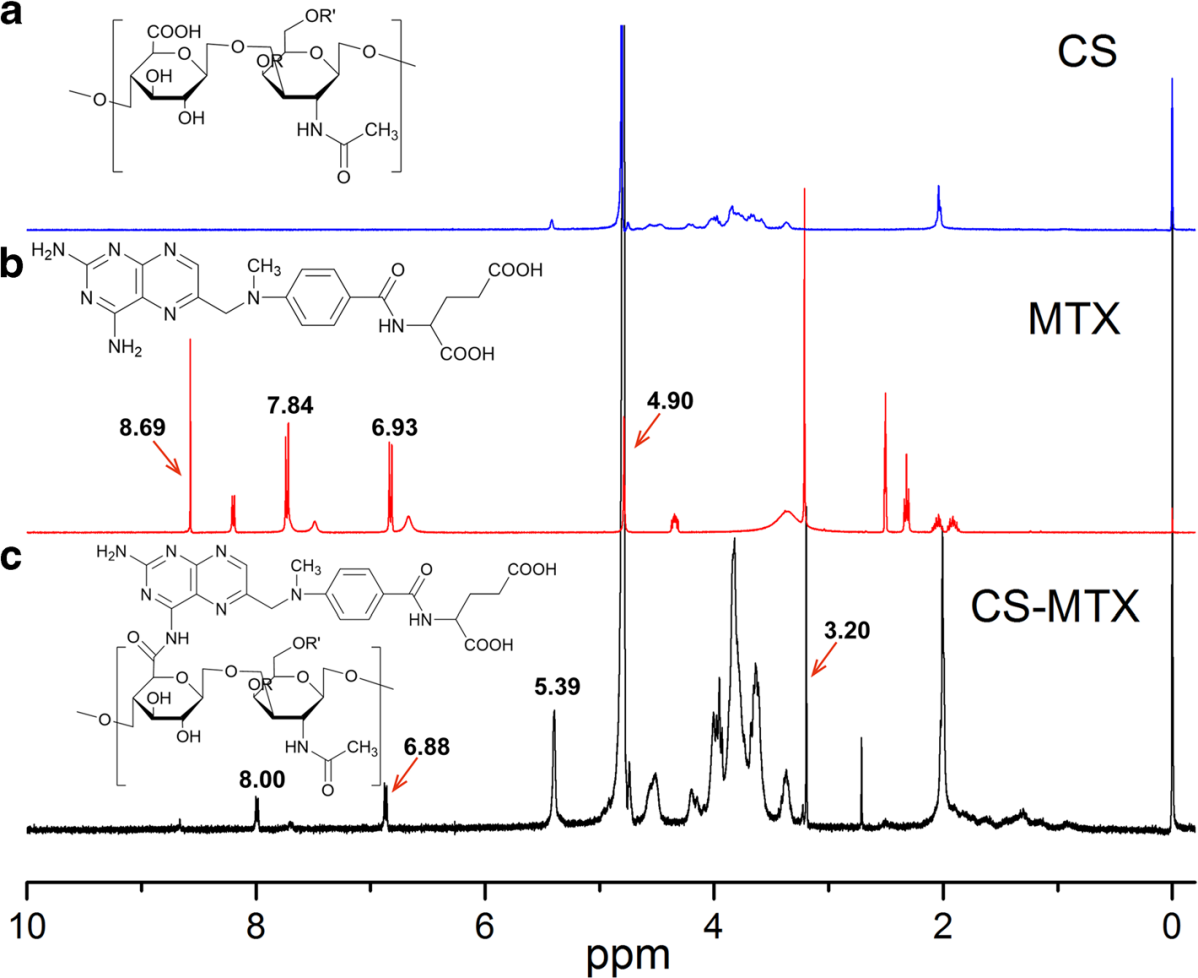
^1^H NMR spectra of CS, MTX, and CS-MTX. **a** ^1^H NMR spectra of CS; CS was dissolved in D_2_O. **b** ^1^H NMR spectrum of MTX; MTX was dissolved in dimethyl sulfoxide-d6. **c** ^1^H NMR spectrum of CS-MTX; CS-MTX was dissolved in D_2_O

Figure 2 shows the ^1^H NMR spectra of CS, MTX, and MTX-CS bioconjugates. The peaks at 6.93 (2H, d, *J* = 10.1 Hz) and 7.84 (2H, d, *J* = 10.1 Hz) can be assigned to the benzoyl group of MTX. The peaks at 4.90 (2H, s) can be assigned to the methylene next to the 2, 4-diamino-6-pteridinyl group, and the peaks at 8.69 (1H, s) can be assigned to the 2, 4-diamino-6-pteridinyl group of MTX as Fig. 2b suggests. The ^1^H NMR of CS-MTX (Fig. 2c) suggested the CS (disaccharide part *δ*_H_ signals were between 3.20 and 5.40, with 5.39 assigned as the anomeric carbon) was successfully attached to MTX (chemical shift of benzoyl group was 8.00 and 6.88, and the methyl group was at 3.20). The NMR also proved that MTX was conjugated to CS.

**Fig. 3:**
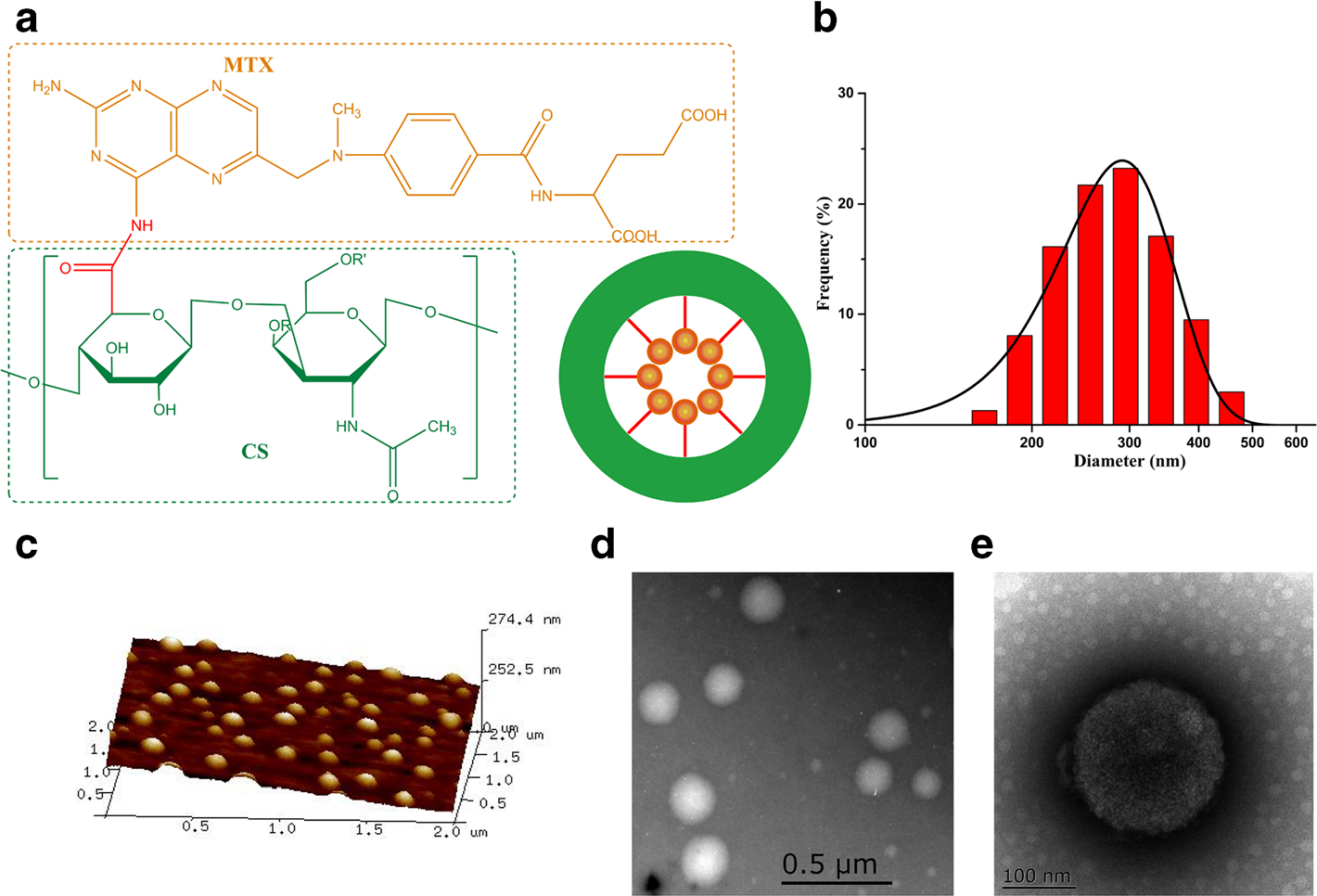
Schematic illustration of the MTX-CS NGs. Dynamic light scattering (DLS), atomic force microscope (AFM), and transmission electron microscopy (TEM) characterization of MTX-CS NGs. **a** Schematic illustration of the MTX-CS NGs. **b** Size of MTX-CS NGs measured by DLS in representative experiments. **c** AFM images of MTX-CS NGs. **d**, **e** TEM images of MTX-CS NGs

To calculate the amount of MTX conjugated to CS, the samples were dissolved in ultrapure water and shaken for 48 h at room temperature. The amount of MTX was determined through the use of a UV-vis spectrophotometer at 309 nm. The amount of MTX was measured by UV-vis spectroscopy. Finally, the calculated amount of methotrexate on MTX-CS NGs was 13.65%. The nanogels formed by encapsulation of hydrophobic MTX molecules by the outside layer of hydrophilic side chains of CS (Fig. 3a). Nanogels were characterized by dynamic light scattering (DLS), atomic force microscope (AFM), and transmission electron microscope (TEM). As shown, DLS data measured the size of all nanogels in the range of 100–400 nm (Fig. 3b). The particle size of the nanoparticles is mainly about 200 nm. AFM image of nanogels confirmed that nanoparticles were well distributed with similar size of about 200 nm and morphology (Fig. 3c). TEM images also shown the size of nanogels were nanospheres with the size in the range of 200–240 nm. The particle size of the nanoparticles is mainly about 200 nm (Fig. 3d, e).

To calculate the amount of MTX conjugated to CS, the samples were dissolved in ultrapure water and shaken for 48 h at room temperature. The amount of MTX was determined through the use of a UV-vis spectrophotometer at 313 nm.

**Fig. 4:**
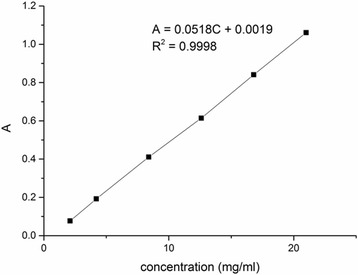
Standard curve of methotrexate UV absorption

The amount of MTX was measured by UV-vis spectroscopy. Firstly, a standard curve of free methotrexate UV absorption was set up (Fig. 4). The relationship between absorbance and free MTX concentration is:

$$ A\kern0.5em =\kern0.5em 0.0518\mathrm{C}\kern0.5em +\kern0.5em 0.0019\ \left({R}^2\kern0.5em =\kern0.5em 0.9998\right) $$

Then, 28.8 mg MTX-CS was dissolved in 1000 ml ultrapure water, and the UV absorption is 0.2055. Finally, the calculated amount of methotrexate on MTX-CS NGs was 13.65%.

### Cytotoxicity of MTX-CS Nanogels

**Fig. 5:**
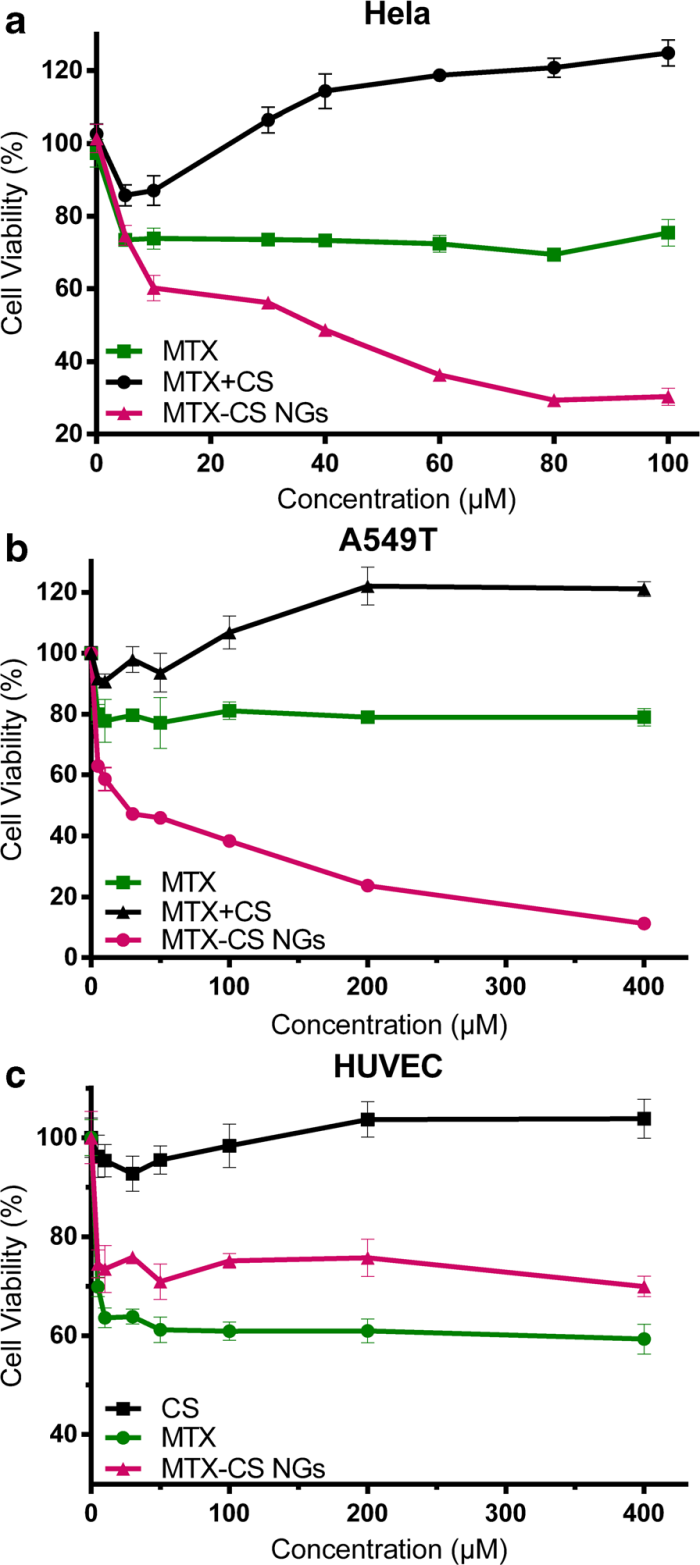
**a** Cellular viability of A549 T in the presence of free MTX, free MTX and CS, and MTX-CS NGs in 48 h. **b** Cellular viability of Hela in the presence of free MTX, free MTX and CS, and MTX-CS NGs in 48 h. **c** Cellular viability of HUVEC in the presence of free MTX, CS, and MTX-CS NGs in 48 h

The in vitro anti-tumor activity of MTX-CS NGs, free MTX, and MTX mixed with CS was analyzed by using both A549T and Hela tumor cell culture. As shown by the MTT assay (Fig. 5), the MTX-CS NGs could significantly reduce the viability of both cancer cells while free MTX did not show any effects at high concentrations. MTX mixed with CS at high concentration even promotes the growth of cancer cells. The viability of Hela was decreased from 73.81% for free MTX to 60.16% for MTX-CS NGs (13.65% decrease in cell viability) at the 10 μM drug concentration (Fig. 5a). Likewise, the A549T cell viability was decreased from 80.23% for free MTX to 46.04% for MTX-CS NGs (34.09% decrease in cell viability) at the 50 μM drug concentration (Fig. 5b). Polysaccharides such as hyaluronic acid are used as a targeting moiety of the drug conjugates or nanoparticles for cancer therapy since it specifically binds to the CD44 receptor [31]. Chondroitin sulfate can also act as a ligand for CD44 receptor [25, 27] which means that CS can promote uptake of MTX-CS NGs by cancer cells and enhance drug efficacy of MTX. In addition, nanoparticles can improve the stability of the drug and control the release of the drugs [32, 33]. All results proved that the anti-tumor activity of MTX-CS NGs was better than free MTX as well as MTX mixed with CS. With increased intracellular delivery efficiency of MTX drug molecules, the targeting selectivity of MTX-CS NGs also improved when compared to the same concentration of free MTX. These results demonstrate that MTX-CS NGs have a better anti-tumor effect than free MTX.

The adverse effect of MTX-CS NGs, free MTX, and CS was analyzed by using HUVEC culture. As shown by the MTT assay (Fig. 5c), the MTX-CS NGs could significantly reduce the side effect while free MTX could significantly reduce the viability of HUVEC. The viability of HUVEC was increased from 63.6% for free MTX to 73.5% for MTX-CS NGs (9.9% increase in cell viability) at the 10 μM drug concentration (Fig. 5c). The cell viability of HUVEC at 400 μM was still 69.95%. The result indicated that MTX-CS NGs could reduce side effect on the normal cell.

### Animal and Experimental Design

**Fig. 6:**
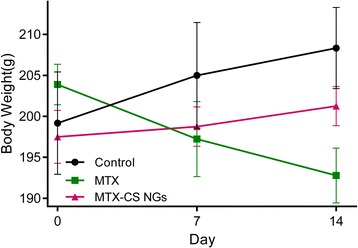
Effects of MTX and MTX-CS NGs on the rat body weight. The day of the first injection was considered as day 0. Saline, MTX (1.25 μmol kg^−1^ day^−1^), and MTX-CS NGs (4.25 mg kg^−1^ day^−1^) were given another day by intraperitoneal injections, respectively, to the corresponding group. Results are expressed as mean ± SEM and analyzed using *t* test. *P < 0.05 compared to the date at day 0

One of the main secondary toxic side effects of MTX used to treat cancer patients is intestinal mucositis, which causes rapid reduction in body weight [34]. We then tested the protective effects of MTX-CS NGs against chemotherapy-induced weight loss in male Sprague-Dawley rats. The survival and weights were monitored for 14 days after injection of saline, free MTX, and MTX-CS NGs. No deaths were found in any of the three groups. An abrupt decrease was observed in the body weight of all the MTX groups (1.25 μmol kg^−1^ day^−1^ for 14 days), clearly indicating that the rats experienced chemotherapy syndrome and chemotherapy-induced damages, resulting in sickness and loss of body weight, while the body weight of rats treated with MTX-CS NGs (4.25 mg kg^−1^ day^−1^ for 14 days) had a slight increase (Fig. 6). The results show that MTX-CS NGs did not cause adverse effects. These findings support the target delivery of MTX to tumor tissue through CD44-CS interaction and reduce cytotoxicity of the MTX drug.

**Fig. 7:**
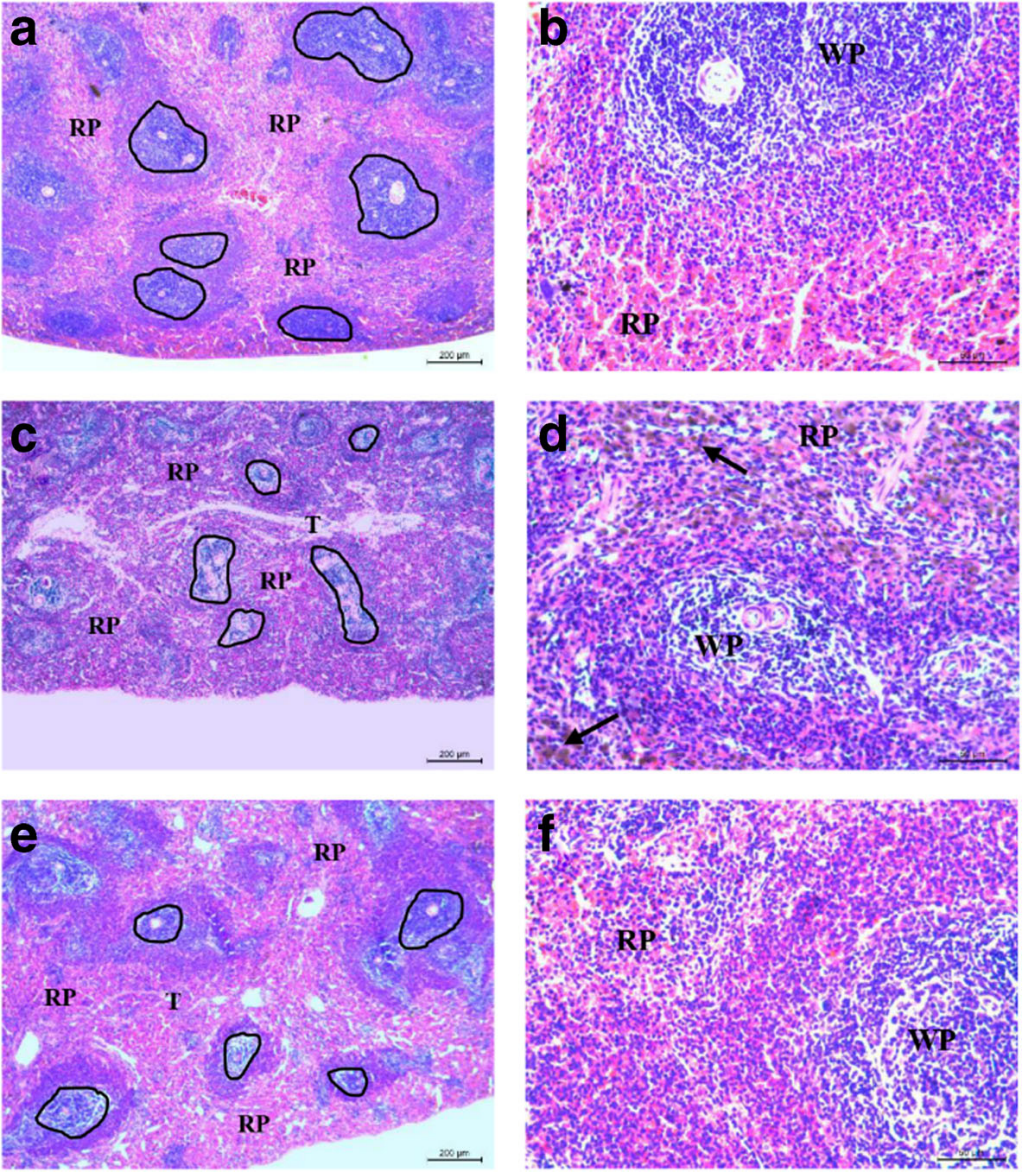
The toxicity effects of MTX and MTX-CS NGs on the rats’ spleen. H&E stained spleen excised from mice following 14 days treatment after seven intraperitoneal injections. **a**, **b** Sections of the control group. **c**, **d** The MTX-treated group. **e**, **f** The MTX-CS NG-treated group

In order to further investigate in vivo toxicity of MTX-CS NGs, a histological analysis of rats’ spleen was performed to determine whether MTX-CS NGs caused tissue damage (Fig. 7). Sections of the control group showed the structure of the normal spleen, composed of white pulp (shapes) and red pulp (RP), with fibrous trabeculae (T) extending into the splenic pulp. The white pulp contains periarterial lymphoid sheaths and splenic follicles and is surrounded by marginal zones, while the red pulp is composed of splenic cords and is separated by splenic sinusoids (Figs. 7a and 7b). The MTX-treated group showed serious narrowing of both the white pulp (black box) and RP. The hemosiderin deposits can also be found in the MTX-treated group (Figs. 7c and 7d). The MTX-CS NG-treated group showed mild narrowing of both the white pulp (shapes) and RP, with no hemosiderin deposits found. Both white pulp and red pulp showed mild narrowing compared with the MTX group (Figs. 7e and 7f). The above results indicated that MTX-CS NGs have little side effects on normal tissue [35, 36].

## Conclusions

In summary, we successfully fabricated self-assembled nanogels for highly efficient anti-tumor drug delivery. The MTX-CS-conjugated nanogels were about 200 nm in size, showing good stability and solubility. MTX-CS NGs showed stronger and more specific cytotoxicity than MTX. In vivo experiments revealed that MTX-CS NGs showed less toxicity than MTX. MTX-CS NGs can improve the anti-tumor effect while reducing the side effects of MTX. Due to their CD44 binding property, chondroitin sulfate-drug conjugates could be a promising and efficient platform for improving the solubility of sparingly soluble drug molecules as well as active and selective targeted delivery to cancer cells and tumor tissues.

## Abbreviations

^1^H NMR: 1H Nuclear magnetic resonance
AFM: Atomic force microscope
CDMT: 2-Chloro-4, 6-dimethoxy-1, 3, 5-triazine
CS: Chondroitin sulfate
DLS: Dynamic light scattering
DMT-MM: 4-(4,6-Dimethoxy-1,3,5-triazin-2-yl)-4-methylmorpholinium chloride
FTIR: Fourier transform infrared
MTT: 3-(4,5-Dimethyl-2-thiazolyl)-2,5-diphenyl-2-H-tetrazolium bromide
MTX: Methotrexate
MTX-CS NGs: Methotrexate-chondroitin sulfate nanogels
NGs: Nanogels
NMM: 4-Methylmorpholine
TEM: Transmission electron microscope
THF: Tetrahydrofuran
UV-vis: Ultraviolet-visible spectroscopy
